# Signal Recognition Particle in Human Diseases

**DOI:** 10.3389/fgene.2022.898083

**Published:** 2022-06-08

**Authors:** Morgana K. Kellogg, Elena B. Tikhonova, Andrey L. Karamyshev

**Affiliations:** Department of Cell Biology and Biochemistry, Texas Tech University Health Sciences Center, Lubbock, TX, United States

**Keywords:** signal recognition particle (SRP), ribosome, disease, cancer, protein targeting and transport, protein sorting, translational control, protein quality control

## Abstract

The signal recognition particle (SRP) is a ribonucleoprotein complex with dual functions. It co-translationally targets proteins with a signal sequence to the endoplasmic reticulum (ER) and protects their mRNA from degradation. If SRP is depleted or cannot recognize the signal sequence, then the Regulation of Aberrant Protein Production (RAPP) is activated, which results in the loss of secretory protein mRNA. If SRP recognizes the substrates but is unable to target them to ER, they may mislocalize or degrade. All these events lead to dramatic consequence for protein biogenesis, activating protein quality control pathways, and creating pressure on cell physiology, and might lead to the pathogenesis of disease. Indeed, SRP dysfunction is involved in many different human diseases, including: congenital neutropenia; idiopathic inflammatory myopathy; viral, protozoal, and prion infections; and cancer. In this work, we analyze diseases caused by SRP failure and discuss their possible molecular mechanisms.

## Introduction

**FIGURE 1:**
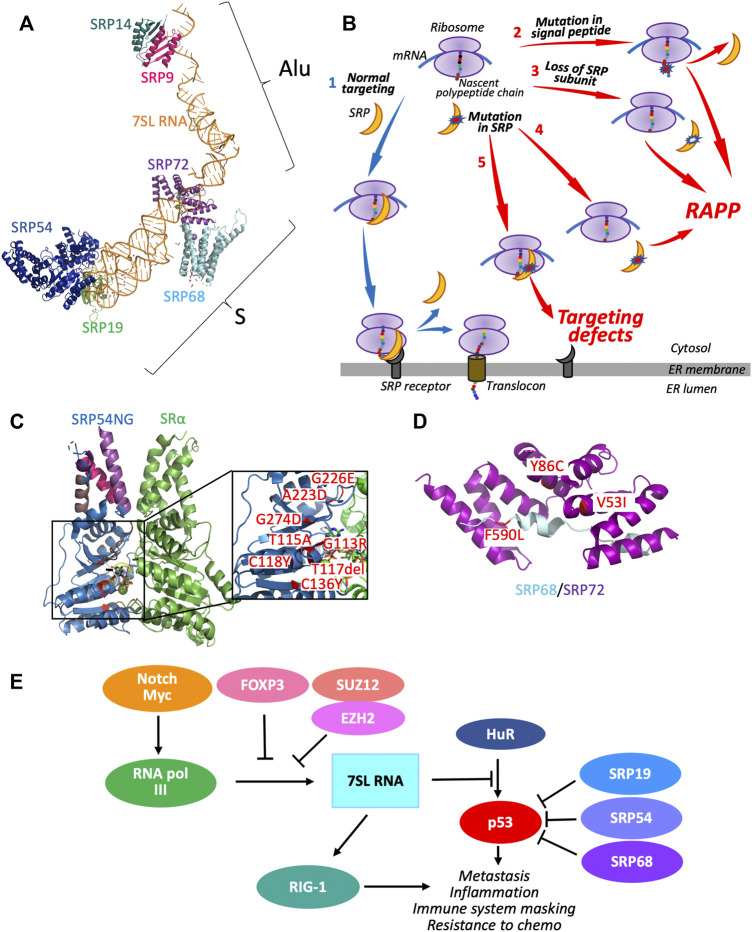
Signal Recognition Particle (SRP) and human diseases. **(A)** SRP molecular model. The picture was prepared using PyMol software (Schroedinger 2020). The SRP protein subunits are assembled on 7SL RNA (orange, from PDB 1RY1 (Halic et al., 2004)), and composed of Alu domain proteins SRP9 (magenta, from PDB 1RY1 (Halic et al., 2004)) and SRP14 (teal, from PDB 1RY1 ( Halic et al., 2004)); and S-domain proteins: SRP72 (purple, from PDB 5WRW (Gao et al., 2017)), SRP68 (light blue, from PDB 5WRV and 4P3F (Grotwinkel et al., 2014; Gao et al., 2017)), SRP19 (green, from PDB 1RY1 (Halic et al., 2004)), and SRP54 (dark blue, from PDB 1MFQ and 1RY1 (Kuglstatter et al., 2002; Halic et al., 2004)). **(B)** SRP cycle and possible dysregulation. Scenario 1–During normal biogenesis, secretory proteins are recognized by SRP (orange crescent) during their synthesis on ribosome (violet ovals). SRP binds N-terminal signal sequence of the nascent polypeptide chain of secretory protein (chain of colored circles), pauses the translation and targets ribosome-nascent chain complex to SRP receptor (dark grey) in ER membrane. Interactions between SRP and SRP receptor lead to transfer of paused ribosomes to the translocon (brown cylinder) followed by release of SRP and continuation of protein synthesis. The nascent chain is translocated through the ER membrane into the lumen for further transport and modifications. Scenarios 2–5 are schematic presentations of events occurring when SRP pathway is defective. Scenario 2–A mutation in the hydrophobic part of the N-terminal signal sequence leads to loss of interactions with SRP and induction of Regulation of Aberrant Protein Production (RAPP) and secretory protein mRNA degradation. Scenario 3–Lack of SRP subunit induces RAPP. Pathology of antibodies against SRP may also lead to interference with functional SRP complex assembly potentially resulting in RAPP. Mutations in SRP subunits may lead to two possible scenarios–if they interfere with SRP binding to nascent chain, they may potentially induce RAPP (Scenario 4); if mutations in SRP subunits do not affect SRP recognition but interfere with SRP binding to SRP receptor, they may impact protein targeting (Scenario 5). Possible dysregulation events, described here, may cause the defects in expression or transport of secretory and membrane proteins in mammalian cells. **(C)** Clinical SRP54 mutations. Mutations in SRP54NG domain (marked in red) shown in proximity to the SRP receptor SRα subunit. **(D)** Clinical SRP68 and SRP72 mutations. Mutations are marked in red, binding domains of SRP68 and SRP72 are in light blue and purple, respectively. Illustrations in **C** and **D** were made by using PyMol software, PDB for SRα is 5L3Q (Wild et al., 2016), coordinates for SRP proteins and references are presented in **(A)**. References for the mutations are presented in the main text. **(E)** SRP proteins and 7SL RNA in cancer regulation. The tumor suppressor protein p53 controls cellular growth by inducing apoptosis if DNA damage is detected. However, p53 function or activity is reduced in cancer cells which allows them to propagate. FOXP3, a master regulator of T-cells, and PRC subunits SUZ12 and EZH2 inhibit the transcription of 7SL RNA, preventing its abnormal expression to downregulate p53. In some cancers, the Notch/Myc transcriptional signal cascade positively regulates RNA pol III (green), which upregulates transcription of 7SL RNA. 7SL RNA binds the 3′-UTR of p53, preventing the interaction with HuR, a positive regulator of p53, and, thus, inhibiting p53 activity. 7SL RNA also activates RIG-1, which stimulates a type-1 interferon pathway. In cervical cancer, SRP proteins SRP19, SRP54, SRP68 abnormally attenuate p53. All these events induce cancer progression.

Many secretory and membrane proteins undergo co-translational targeting to the endoplasmic reticulum (ER) governed by the signal recognition particle (SRP). SRP is a ribonucleoprotein consisting of six protein subunits arranged on a long noncoding RNA called 7SL RNA (Figure 1A). SRP is divided into two major domains: the Alu (7SL RNA Alu region with SRP9 and SRP14 proteins) which functions in elongation arrest; and the S or signal recognition domain (7SL RNA S region with SRP19, SRP54, SRP68, and SRP72 proteins). SRP targets proteins using a series of events called the SRP cycle (Figure 1B Scenario 1). First, SRP recognizes signal sequences of secretory proteins upon their exposure from the ribosome during translation, leading to elongation arrest. SRP-ribosome complexes then move to the SRP receptor on the ER membrane. Finally, SRP hands over the ribosome to the SEC61 translocon and hydrolyzes GTP. SRP leaves the complex, translation elongation resumes, and the nascent polypeptide chain is translocated through the translocon into the ER lumen. The SRP cycle is reviewed in detail in (Kellogg et al., 2021). It is estimated that more than 30% of eukaryotic proteins are secretory or membrane proteins (Uhlen et al., 2015), and many of them may be targeted by SRP. Thus, defects in SRP biogenesis and mutations in SRP subunits may have pathological effects on a large number of these substrates leading to human diseases.

When SRP cannot recognize the signal sequence due to mutations decreasing its hydrophobicity, it induces a quality control mechanism called the Regulation of Aberrant Protein Production (RAPP) (Karamyshev et al., 2014; Karamyshev and Karamysheva 2018) (Figure 1B, Scenario 2). We established that RAPP is pathologically activated by signal sequence defects associated with a number of human diseases: aspartylglucosaminuria (aspartylglucosaminidase), Norrie disease (Norrie disease protein), hypoparathyroidism (parathyroid hormone), frontotemporal lobar degeneration (granulin), and many others (Pinarbasi et al., 2018; Karamysheva et al., 2019; Tikhonova et al., 2019; Karamyshev et al., 2020). RAPP results in mRNA degradation of secretory proteins that have mutations reducing hydrophobicity of signal sequences demonstrating a novel type of molecular basis for multiple diseases (Karamyshev et al., 2020). Interestingly, we discovered that the loss of the SRP54 subunit of SRP also induces RAPP (Karamyshev et al., 2014; Pinarbasi et al., 2018; Tikhonova et al., 2019) (Figure 1B, Scenario 3). These observations suggest that mutations in SRP54 or other subunits that affect their functions may also trigger RAPP (Figure 1B Scenario 4). Dysfunction may also occur when SRP cannot bind to the SRP receptor (Figure 1B Scenario 5), and therefore the ribosome cannot reach the translocon. In this work, we analyze and discuss SRP involvement in multiple types of diseases, including hematological disorders (congenital neutropenia, anemia, others), auto-immunity, neurological diseases, cancer, and possible molecular mechanisms.

### Signal Recognition Particle in Hematological Diseases

SRP54 and SRP72 harbor autosomal-dominant mutations, which cause different types of hematological diseases (Kirwan et al., 2012; Bellanne-Chantelot et al., 2018). Patients with SRP54 heterozygous mutations exhibit neutropenia (low number of neutrophils), transient moderate anemia, frequent infections, and skeletal abnormalities. Eight different SRP54 G-domain autosomal dominant mutations were identified, as shown in Figure 1C (Burwick et al., 2012; Carapito et al., 2017; Bellanne-Chantelot et al., 2018; Juaire et al., 2021; Schurch et al., 2021). Some of these mutations (about 13% of patients) mimic the pathology associated with Schwachman-Diamond syndrome. Regardless of the mutation locations in G-domain they dramatically reduce granulocyte differentiation (Bellanne-Chantelot et al., 2018). Three mutations have been studied in detail: T115A, T117del, and G226E; they affect SRP54 protein stability and GTPase activity leading to ER stress, UPR activation with insufficient XBP1 splicing and aberrant protein translation (Carapito et al., 2017; Bellanne-Chantelot et al., 2018; Juaire et al., 2021; Schurch et al., 2021). It was also shown that SRP54 mRNA expression was reduced in patients’ bone marrow (Carapito et al., 2017). Although the exact molecular mechanisms behind congenital neutropenia caused by mutations in SRP54 is still not completely understood, they may be connected with defects in protein targeting caused by reduced GTPase activity; or in some cases by inducing RAPP when SRP54 expression is affected, though there is no experimental data so far. Each studied mutation causes a similar pathology–yet the mechanism may be different in each of the eight mutations. The mutation G226E may interfere with packing necessary for interaction between SRP54 and SRα.

Abnormalities in SRP72 also cause a bone marrow pathology. SRP72 heterozygous mutant patients exhibit deafness and aplastic anemia, where immature bone marrow cells become deformed and non-functional (Kirwan et al., 2012). The SRP72 mutation T355Kfs^∗^19 causes synthesis of a truncated protein, inhibiting the binding of SRP72 to 7SL RNA and SRP68. This truncation likely affects export from the nucleus, ribosome binding, and the ability to target proteins to the ER. The R207H mutation may disrupt the protein binding interface between SRP72 and SRP68 or SRP54 impacting the targeting of proteins to the ER. Also, it is still unknown why R207H and T355Kfs^∗^19 mutations cause milder forms of aplasia and myelodysplasia in mice than in humans (D'Altri et al., 2019). Recently, SRP68 with A50Ffs^∗^52 has also been shown to cause congenital neutropenia, and likely causes a loss in binding 7SL RNA and subsequent protein targeting problems (Schmaltz-Panneau et al., 2021). The SRP72 mutations V53I and Y86C, and the SRP68 mutation F590L (as shown in Figure 1D) also inhibit targeting of proteins to the ER and have been observed in cancer (Gao et al., 2017).

### Signal Recognition Particle in Autoimmunity

Autoimmunity also has a link to SRP and protein targeting. Anti-SRP antibodies are prevalent in immune-mediated necrotizing myositis (IMNM), an autoimmune rheumatological disease with a incidence of 17–45% of all patients with idiopathic inflammatory myopathies (IIM) (Watanabe et al., 2016). IIM itself has an incidence of 2 in 100,000 (Smoyer-Tomic et al., 2012). IMNM (or anti-SRP IIM) clinically presents with myopathy, endomysial fibrosis, and necrosis (Nakashima 2018; Christopher-Stine et al., 2022). Anti-SRP antibodies are associated with chronic disease and aggressive and severe myopathy with little control with immunosuppressants and glucocorticoids (Targoff 2021; Christopher-Stine et al., 2022). Only four antibodies against SRP have been associated with necrotizing myopathy–anti-SRP19, anti-SRP54, anti-SRP72, and anti-7SL RNA (Satoh et al., 2005; Romisch et al., 2006; Apiwattanakul et al., 2016; Wang et al., 2016). Anti-SRP19 auto-antibodies are the most common cause of necrotizing myopathy and are suspected to be a factor in the pathogenesis of anti-SRP IIM (Wang et al., 2016).

The mechanism behind the pathogenesis of anti-SRP antibody linked IMNM is largely unknown. It has been demonstrated that anti-SRP54 antibodies significantly impair protein targeting to ER *in vitro* (Romisch et al., 2006). It is still enigmatic how SRP subunits are subject to immune system destruction. Anti-SRP antibodies are involved with the complement cascade C3 convertase and paraneoplastic syndrome, hinting that presentation of SRP subunits (or fragments thereof) to CD5^+^ B-cells or CD4^+^ T-cells may be a factor in pathogenesis (Utz et al., 1998; Acciavatti et al., 2013; Bergua et al., 2019). However, it is still unclear whether phosphorylation, caspase cleavage, or some other mechanism mediates autoimmunity to SRP.

Lastly, SRP19 is involved in another rheumatological disease called Kashin-Beck. SRP19 is upregulated in Kashin-Beck disease, an osteochondropathy that causes the death of cartilage cells (Zhang et al., 2018). Dysfunction could be due to the dysregulation of SRP19; either too much (in Kashin-Beck) or too few (in anti-SRP IIM) causes diseases.

### Signal Recognition Particle in Neurological, Neurodegenerative, and Infectious Diseases

Defects in signal sequence recognition and protein targeting are also connected with neurodegenerative diseases. Thus, mutations in granulin signal sequence associated with frontotemporal lobar degeneration inhibit interaction with SRP54 leading to granulin mRNA degradation implicating pathological RAPP activation in the disease (Pinarbasi et al., 2018; Karamysheva et al., 2019). SRP also plays a role in alpha-synuclein biogenesis and in Parkinson’s disease, although the mechanism is still unknown (Hernandez et al., 2021). There are indications that upregulated SRP9 causes mesial temporal lobe epilepsy (Hessel et al., 2014). SRP9 and SRP14 are also major components of stress granules, and interact with neuronal BC200 (Xiao et al., 2012; Berger et al., 2014). BC200 is involved in translational regulation in dendrites (Khanam et al., 2007; Mus et al., 2007; Xiao et al., 2012; Booy et al., 2021), and dysregulation of BC200 has been shown in Alzheimer’s patients (Mus et al., 2007). SRP9 has also been shown to be associated with plaque formation in Alzheimer’s disease (Li and De Muynck 2021), suggesting there could be a causal link between BC200, SRP9, and the pathogenesis of Alzheimer’s disease.

Additionally, 7SL RNA, the scaffold of SRP, is connected to viral, protozoal, and prion infections. HIV recruits and processes 7SL RNA in cells and packages it in virions as a remnant without the SRP54 protein component (Keene et al., 2010). The 7SL RNA interacts with Gag, a viral membrane protein family that mediates assembly, maturation, and release of virions and nucleocapsid proteins (Itano et al., 2018). 7SL RNA is also an indicator of an active trypanosomiasis infection and an attractive target for inhibition (Chiweshe et al., 2019). Lastly, the prion PrP interacts and depletes 7SL through RNA-binding domains in scrapie, a sheep neurodegenerative disease (Gomes et al., 2008; Lathe and Harris 2009).

### Signal Recognition Particle in Cancer

SRP is a factor implicated in cancer progression. Cancer is marked by ten hallmarks: sustained proliferation, evading growth suppression, avoidance of the immune system, cell immortality, inflammation, invasion and metastasis, angiogenesis, mutability, anti-apoptotic measures, and deregulation of cell energetics (Hanahan and Weinberg 2011). Cancer-related mutations and dysregulation of SRP in various types of cancer have caused a number of these hallmarks.

One of the most common mechanisms of cancer is to deactivate the tumor suppressor p53; this occurs through mutations, deletions, or attenuating the protein. p53 suppression protects cancer cells from destruction by apoptosis and evades the growth checkpoint between G1 and S phases. SRP19, SRP54, and SRP68 attenuate the p53 protein. It was shown in cervical cancer that the SRP subunits SRP19, SRP54, and SRP68 interact with p53 and decrease the number of copies of p53 available (Abdelmohsen et al., 2014). Additionally, 7SL RNA, the RNA pol III transcribed structural backbone of SRP, directly interacts with the 3’ UTR of p53 through putative binding sites that out-competes binding by HuR, a positive regulator of p53 (Abdelmohsen et al., 2014). However, tumor FOXP3 indirectly activates p53 by suppressing 7SL activity (Yang et al., 2016), suggesting a mechanism of interplay between the three proteins. Further studies have shown breast cancer Polycomb repressive complex (PRC), with subunits EZH2 and SUZ12, suppresses 7SL transcription by H3K27 triple methylation at the 7SL promoter (Liu et al., 2015). Figure 1E illustrates the intricate relationship between SRP proteins and 7SL RNA and cancer.

SRP dysfunction in cancer can also cause invasion and metastasis, promotes inflammation, avoids immune system destruction, and has anti-apoptotic measures. When SRP9 and SRP14 are absent from the 7SL RNA Alu-domain in triple-negative breast cancer, it increases metastasis and activates RIG-1, a pattern recognition receptor usually reserved for viral infections (Nabet et al., 2017). RIG-1 causes an interferon response, which increases inflammation and metastasis in breast cancer, and drives therapy resistance (Boelens et al., 2014). 7SL RNA’s role in other types of cancer is unknown, and, likely, the suppression of p53 by 7SL is also driving tumorigenesis in triple-negative breast cancer.

SRP subunits could be predictive markers of cancer, though only SRP9 and SRP14 have been investigated as prognostic tools. Colorectal cancer upregulates adenosine-to-inosine RNA editing enzymes which edit the mRNA of SRP9 causing upregulation and indicating SRP9 can be used as a prognostic marker in these cancers (Rho et al., 2008; Lee et al., 2017). SRP9 has been discovered as an aberrant fusion gene with epoxide hydrolase 1 in Non-Hodgkin’s lymphoma (Matsumoto et al., 2021). SRP14 is a diagnostic marker in hepatocellular cancer (Li et al., 2021).

## Discussion

Multiple human diseases are associated with SRP defects. Different disorders involve deficiencies/mutations in distinct SRP protein subunits or in its non-coding RNA. Defects or association with diseases are demonstrated for all SRP subunits suggesting multiple molecular mechanisms for different disorders. Some of them may be associated with inefficiency of protein targeting and transport as a consequence of defects in GTPase activity, decrease in association with the SRP receptor and translocon, or other disruptions. Other diseases may be associated with reduced recognition of signal sequences by SRP, thus triggering the pathological activation of the RAPP pathway and its following mRNA degradation of secretory proteins. While all SRP subunits are important for the complex functioning, the SRP54 subunit interacts with signal sequences directly, thus, aberrant SRP54 is the most likely candidate for activation of RAPP. Since congenital neutropenia involves mutations in SRP54, it is possible that one of the mechanisms of disease is the activation of RAPP at least in part (Figure 1B Scenario 4). However, we would like to acknowledge that RAPP activation is still speculative since the mutations are not directly localized in the signal recognition domain of SRP54. In IMNM, antibodies are attacking the SRP proteins themself. Thus, if SRP54 is depleted because of degradation by the immune system, then RAPP could be activated (Figure 1B Scenario 3). Depletion of SRP19 may affect the binding interface on 7SL RNA for SRP54, which could induce RAPP and cause the depletion of secreted proteins in the extracellular matrix. 7SL involvement in disease is more esoteric; it may be activating RAPP with its dysregulation in SRP complex, but 7SL RNA also has SRP-independent functions and could be causing completely different mechanisms of disease (Talhouarne and Gall 2018).
